# Supplementary material for: Aftershocks are fluid-driven and decay rates controlled by permeability dynamics
Source: Nat Commun. 2020 Nov 13;11:5787. doi: 10.1038/s41467-020-19590-3 (PMC7666175; doi:10.1038/s41467-020-19590-3)
Supplement: Supplementary file 1 — Supplmentary Information [file 41467_2020_19590_MOESM1_ESM.pdf]

Supplementary Information for

Aftershocks are fluid-driven and decay rates controlled by permeability dynamics

by
Stephen A. Miller

Center for Hydrogeology and Geothermics (CHYN)
University of Neuchâtel
2000 Neuchâtel, Switzerland

Email: stephen.miller@unine.ch

1.0 Supplementary Tables

Supplementary Table 1. Comparison of different earthquakes and their aftershocks

Earthquake	Date	FocalMech.	Mw	Hypocenter (km)	M≥4 foreshocks?	M≥4 Aftershocks (first 24 h)	Total M≥4 Aftershocks (1st 3 weeks)
Peru	26.5.2019	Normal	8.0	78	No	0	0
Mexico	17.9.2017	Normal	7.1	48	No	0	0
Jamaica	28.1.2020	Strike-slip	7.7	15	No	21	26
Kunlun	3.11.2002	Strike-slip	7.8	10	No	7	12
Denali	14.11.2001	Strike-slip	7.9	4.2	Yes (3)	90	157
Ridgecrest	4.7.2019	Strike-slip	7.1	8	Yes (26)	67	82
Puerto Rico	7.2.2020	Normal	6.4	7.4	Yes (10)	29	87
Landers	28.6.1992	Strike-slip	7.3	0.1	Yes (1) Swarms**	30*	50
Northridge	17.1.1994	Thrust	6.7	14	Yes (2 clusters M<4)	28	54
Hector Mine	16.10.1999	Strike-slip	7.1	7.4	Yes (swarms)[1]	34	55

*not including Big Bear,**Chen/Shearer. Source http://earthquake.usgs.gov

2.0 Supplementary Figures

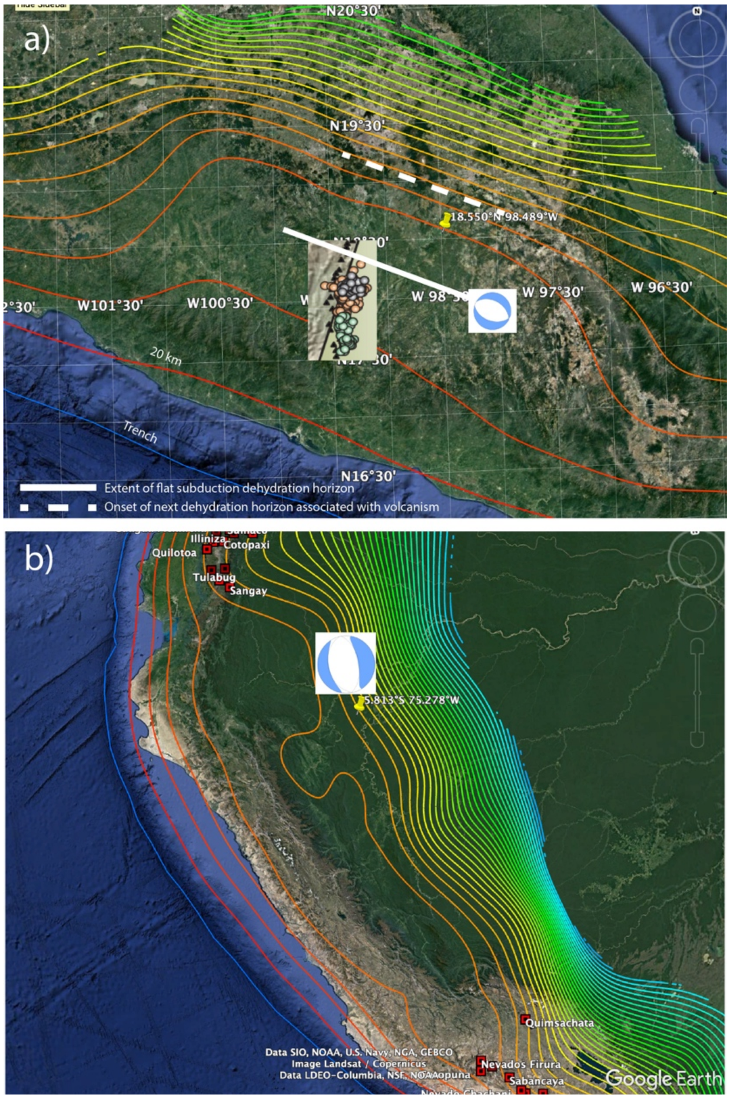

Supplementary Figure 1. Examples of aftershock-free great and major earthquakes. (a,b) Both the Mexico and Peru hypocenters although associated with slab subduction, occurred far from the trench, and were located where flat subduction transitions to steeply dipping slab penetration into the mantle. Their normal faulting focal mechanisms are consistent with flexural stresses associated with this transition.

The volcanic arcs in South America roughly coincide with the 100 km iso-depth (Figure 1b) and correlates with the antigorite$\rightarrow$enstatite+H_2O dehydration reaction that reduces the melting temperature of the overlying hot mantle rocks. Peru is devoid of a volcanic arc and this is largely attributed to flat subduction[2] that extends up to 300 km along the 80 km iso-depth. Presumably this results in a pressure-temperature (P-T) path that avoids this important antigorite$\rightarrow$ enstatite phase transition. Modeling of slab PT paths for Peru[3] suggest that many dehydration boundaries are crossed at shallow depths, which might relate to massive ore deposits in Peru[4] indicating vast hydrothermal fluid flow in the geologic past, but these are in the coastal mountains about 500 km west of the earthquake hypocenter. The lack of any volcanoes in Peru, nor detection of non-volcanic tremor (NVT) to date provides strong evidence that fluids are never released, and that the M8 earthquake occurred in dry lithosphere. It is also possible

that the lack of the fluid above the subduction interface results from complex fluid paths driven by tectonic stresses in the overriding plate[5].

Flat subduction also occurs in Mexico. Extensive seismic surveys of NVT[6] show NVT clustering in the flat subduction region, but NVT terminates about 80 km trench-ward from the earthquake hypocenter (Supplementary Figure 1a). Separate studies of the thermal and petrological structure of the subducting Cocos plate [7] show that the final dehydration reaction in the flat subduction region correlates with these NVT locations. Subsequent dehydration horizons occur as the plate descends into the mantle, which correlates with the onset of volcanism, but this occurs at a depth of 100 km (50 km deeper than the M7.1 earthquake hypocenter), and the nearest volcano (Popocatepetl) lies 55 km to the NE of the hypocenter. This means that the Mexico earthquake initiated in-between two dehydration horizons and thus the lithosphere that ruptured was fluid-poor.

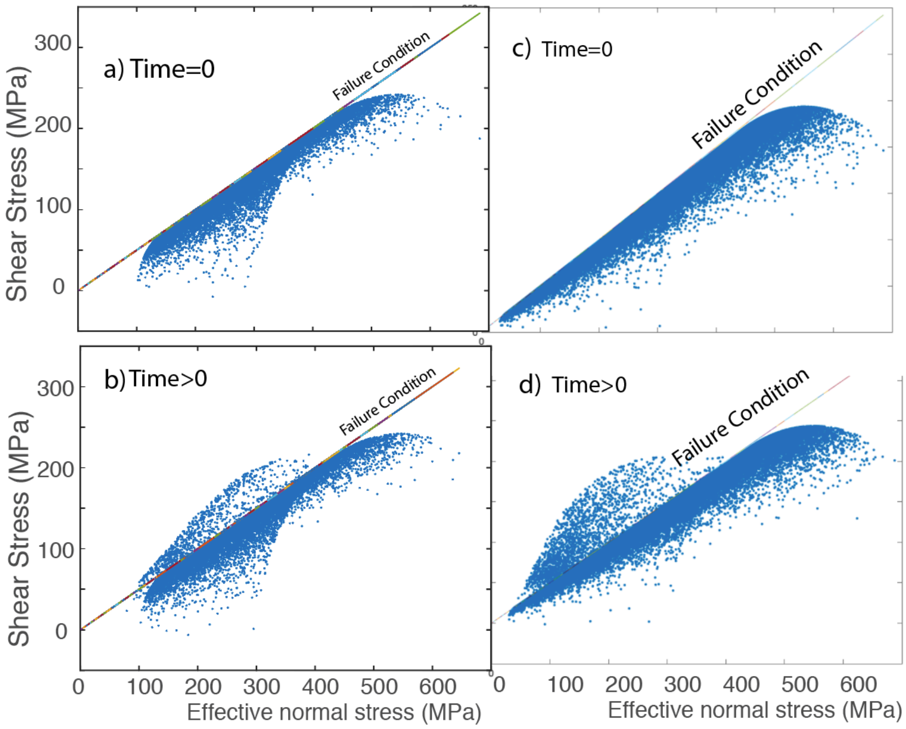

Supplementary Figure 2. Numerical Aftershocks. Stress state at time=0 for **a)** Northridge and **c)** all other simulations**.** Numerical "aftershocks" are counted and located every time the state of stress (blue dot) reaches or exceeds the frictional failure condition **(b-d)**. Aftershocks are counted in the model from t≥1 day to respect short term dynamic and elastic processes operating that are not modelled. Further model details found elsewhere[8]

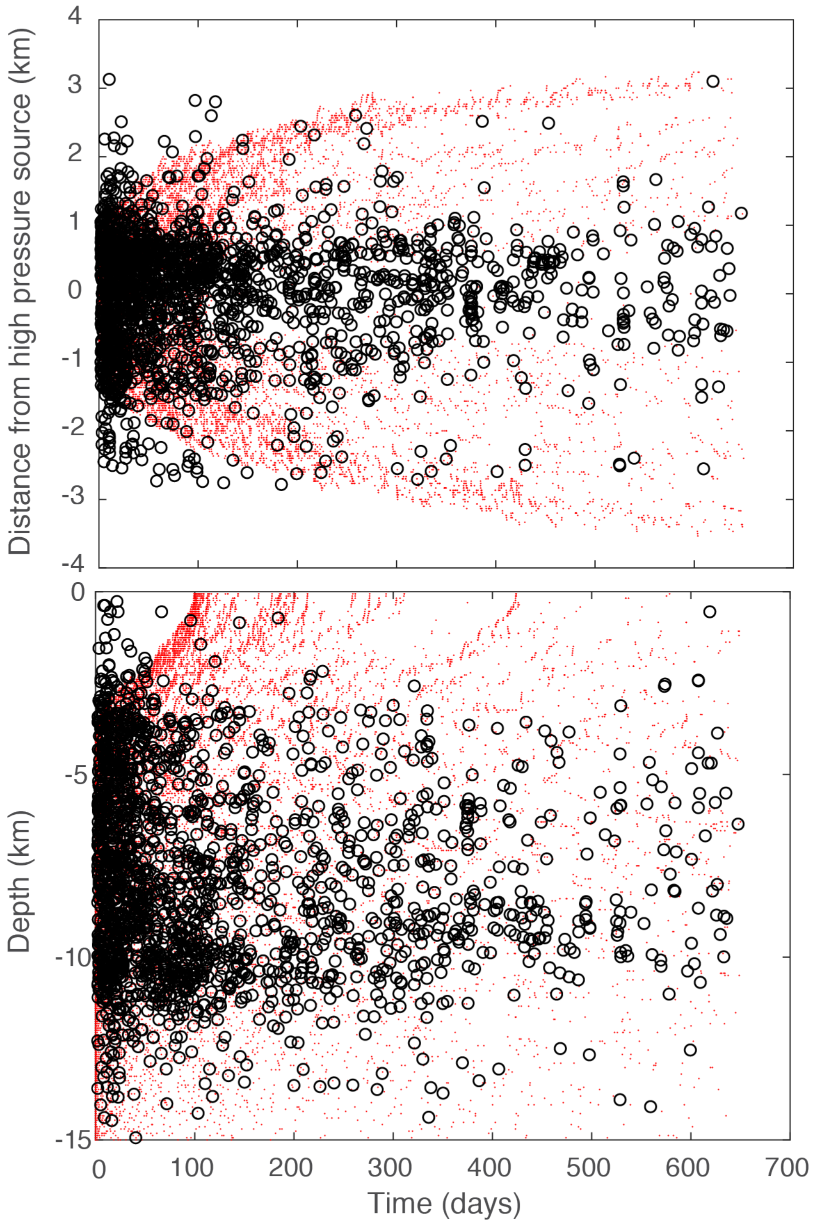

Supplementary Figure 3. Space-time evolution on data and model. One objection to this model is that migration is not observed but is always part of a diffusion model. Recent observations from Ridgecrest[9], using results from high-density seismic arrays, do indicate migration and potentially rendering moot this objection. Nevertheless, this can be investigated for the earthquakes in this study by comparing model and measured hypocenters in the x-t and z-t plane. The model shows a subtle diffusion front in the x-t plane, as expected, with less obvious diffusion in the z-t plane because events occur in the model throughout the depth. A diffusion front in the data is not obvious, but this may be a result of insufficient seismic coverage of these long-passed events. The model predicts that, given sufficient seismic coverage, migration will be found for all rich aftershock sequences.

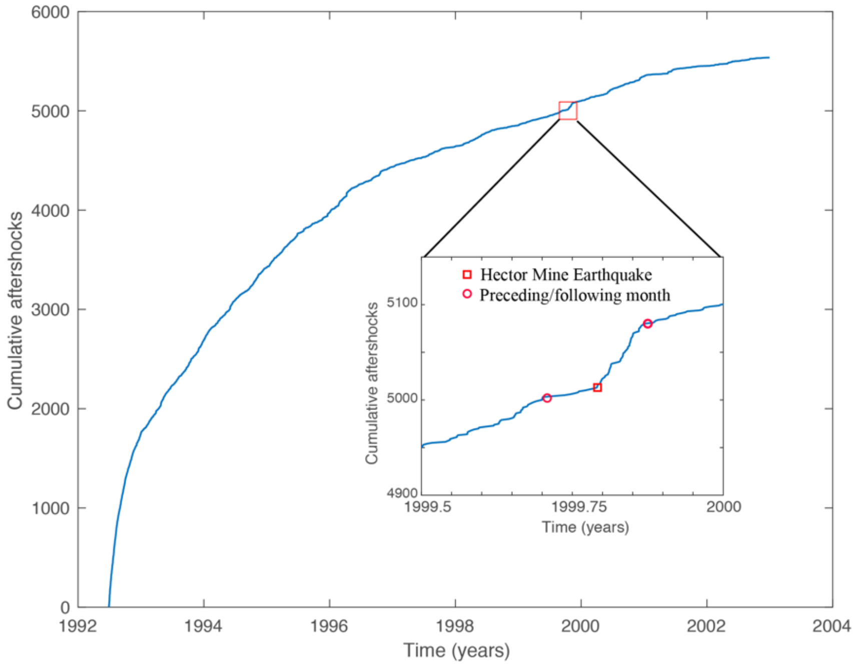

Supplementary Figure 4. Response of the Stepover to the Hector Mine earthquake where more than 70 events were triggered, clustering at around 12 km depth, a response indicative of hydrothermal conditions at depth in the Stepover[10].

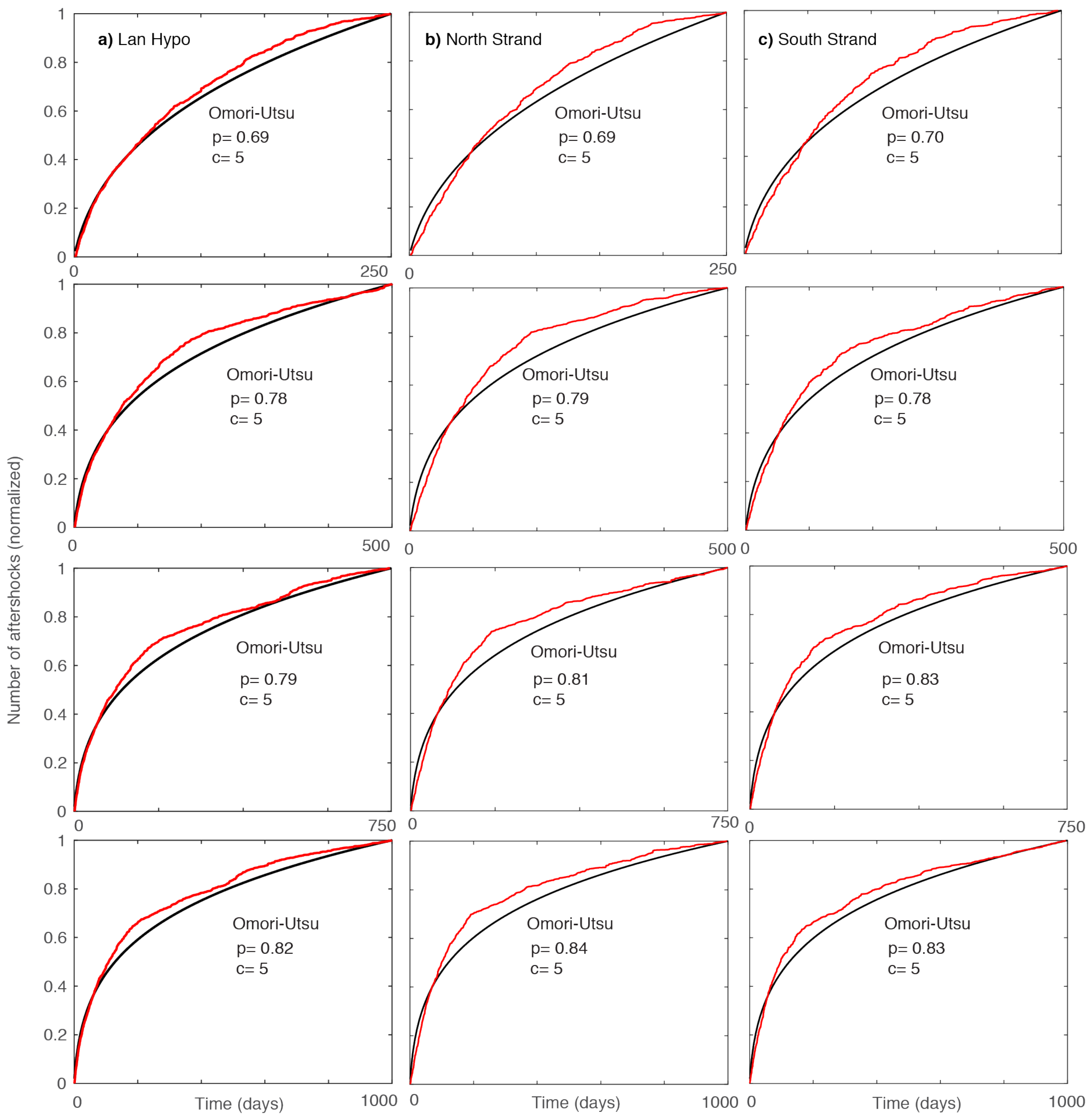

Supplementary Figure 5. Data fits to Omori-Utsu Law. Fits to Omori-Utsu at different time windows for column **a)** Lan Hypo, column **b)** North Strand, and column **c)** South Strand.

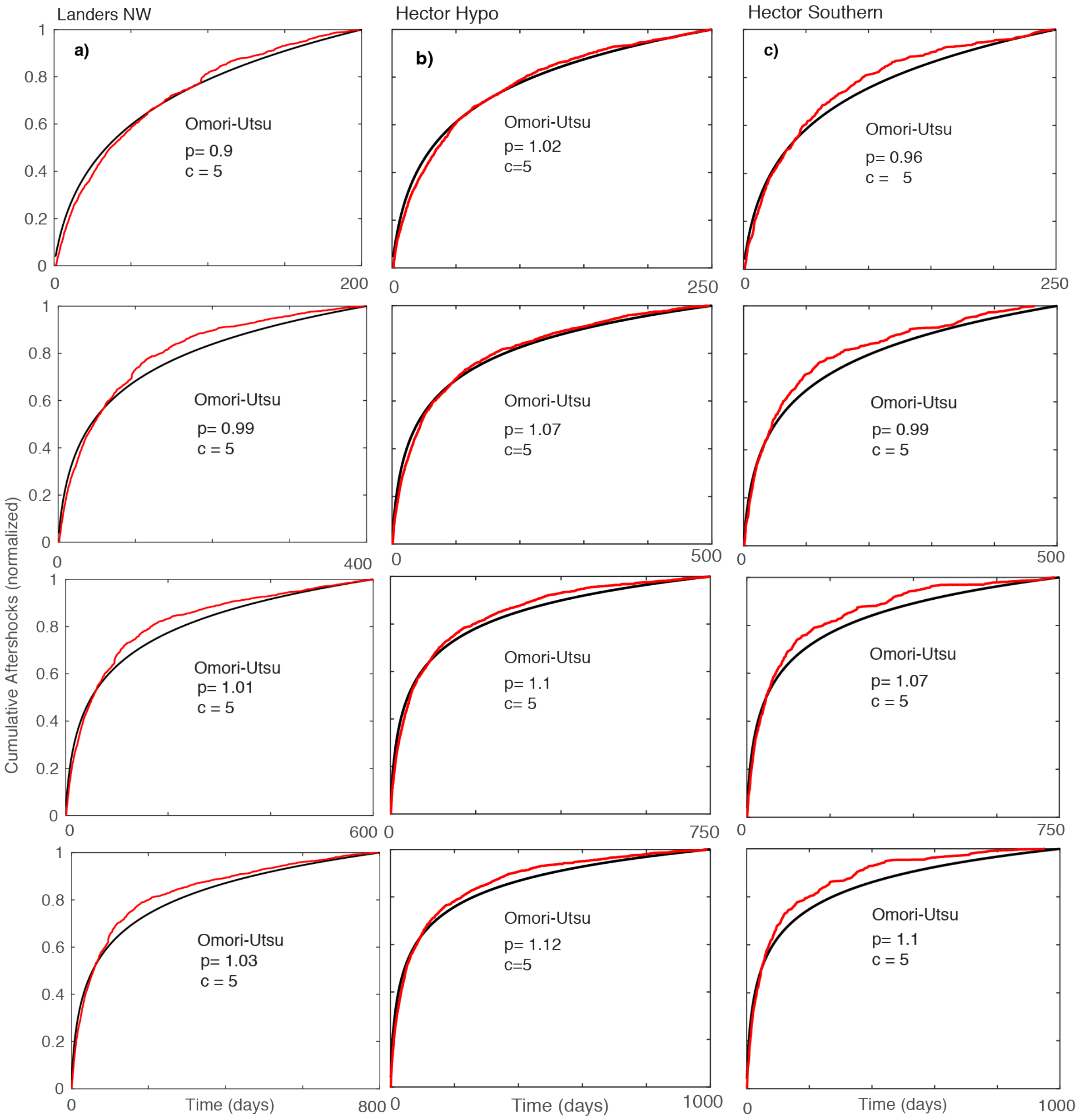

Supplementary Figure 6. Data fits to Omori-Utsu Law. Fits to Omori-Utsu at different time windows for column **a)** Landers NW, column **b)** Hector Hypo, and column **c)** Hector Southern.

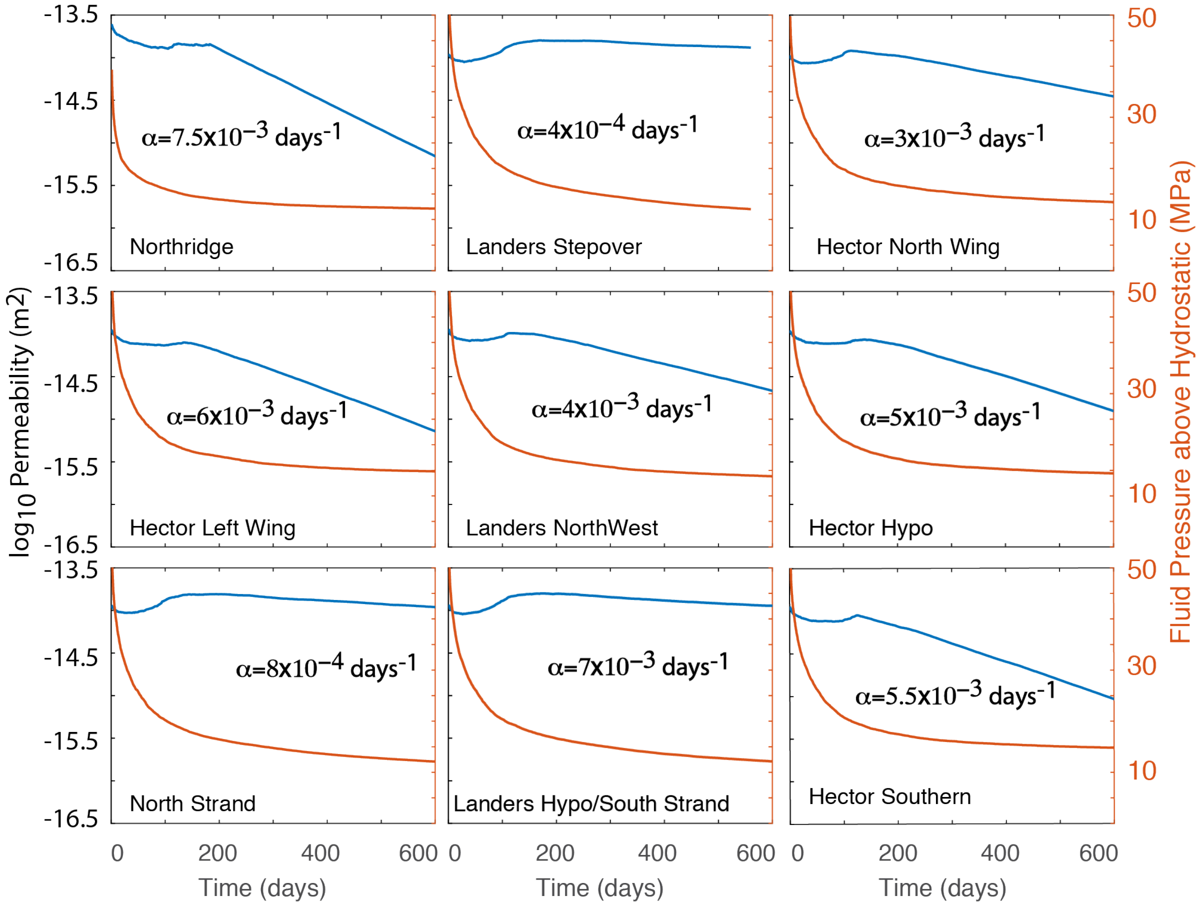

Supplementary Figure 7. Compendium of pressure-permeability histories for all simulations.

Supplementary References:

1 Chen, X., Shearer, P. & Abercrombie, R. Spatial migration of earthquakes within seismic clusters in Southern California: Evidence for fluid diffusion. *Journal of Geophysical Research: Solid Earth* **117** (2012).

2 Gutscher, M. A., Spakman, W., Bijwaard, H. & Engdahl, E. R. Geodynamics of flat subduction: Seismicity and tomographic constraints from the Andean margin. *Tectonics* **19**, 814-833 (2000).

3 Syracuse, E. M., van Keken, P. E. & Abers, G. A. The global range of subduction zone thermal models. *Physics of the Earth and Planetary Interiors* **183**, 73-90, doi:10.1016/j.pepi.2010.02.004 (2010).

4 Rosenbaum, G. *et al.* Subduction of the Nazca Ridge and the Inca Plateau: Insights into the formation of ore deposits in Peru. *Earth and Planetary Science Letters* **239**, 18-32 (2005).

5 Menant, A., Angiboust, S. & Gerya, T. Stress-driven fluid flow controls long-term megathrust strength and deep accretionary dynamics. *Scientific reports* **9**, 9714 (2019).

6 Husker, A. L. *et al.* Temporal variations of non-volcanic tremor (NVT) locations in the Mexican subduction zone: Finding the NVT sweet spot. *Geochemistry, Geophysics, Geosystems* **13** (2012).

7 Manea, V. C. & Manea, M. Flat-slab thermal structure and evolution beneath central Mexico. *Pure and Applied Geophysics* **168**, 1475-1487 (2011).

8 Miller, S. A. Modeling enhanced geothermal systems and the essential nature of large-scale changes in permeability at the onset of slip. *Geofluids* **15**, 338-349 (2015).

9 Shelly, D. R. A High-Resolution Seismic Catalog for the Initial 2019 Ridgecrest Earthquake Sequence: Foreshocks, Aftershocks, and Faulting Complexity. *Seismological Research Letters* (2020).
10 Miller, S. A. & Mazzini, A. More than ten years of Lusi: A review of facts, coincidences, and past and future studies. *Marine and Petroleum Geology* **90**, 10-25 (2017).
